# Enterococcal bloodstream infections in critically ill patients with COVID-19: a case series

**DOI:** 10.1080/07853890.2021.1988695

**Published:** 2021-10-12

**Authors:** Daniele Roberto Giacobbe, Laura Labate, Stefania Tutino, Federico Baldi, Chiara Russo, Chiara Robba, Lorenzo Ball, Silvia Dettori, Anna Marchese, Chiara Dentone, Laura Magnasco, Francesca Crea, Edward Willison, Federica Briano, Denise Battaglini, Nicolò Patroniti, Iole Brunetti, Paolo Pelosi, Matteo Bassetti

**Affiliations:** a Department of Health Sciences (DISSAL), University of Genoa, Genoa, Italy; b Clinica Malattie Infettive, San Martino Policlinico Hospital – IRCCS for Oncology and Neurosciences, Genoa, Italy; c Department of Surgical Sciences and Integrated Diagnostics (DISC), University of Genoa, Genoa, Italy; d Anaesthesia and Intensive Care, San Martino Policlinico Hospital – IRCCS for Oncology and Neurosciences, Genoa, Italy; e Microbiology Unit, San Martino Policlinico Hospital – IRCCS for Oncology and Neurosciences, Genoa, Italy; f Department of Medicine, University of Barcelona, Barcelona, Spain

**Keywords:** *Enterococcus*, VRE, BSI, COVID-19, SARS-CoV-2

## Abstract

**Background:**

An unexpected high prevalence of enterococcal bloodstream infection (BSI) has been observed in critically ill patients with COVID-19 in the intensive care unit (ICU).

**Materials and methods:**

The primary objective was to describe the characteristics of ICU-acquired enterococcal BSI in critically ill patients with COVID-19. A secondary objective was to exploratorily assess the predictors of 30-day mortality in critically ill COVID-19 patients with ICU-acquired enterococcal BSI.

**Results:**

During the study period, 223 patients with COVID-19 were admitted to COVID-19-dedicated ICUs in our centre. Overall, 51 episodes of enterococcal BSI, occurring in 43 patients, were registered. 29 (56.9%) and 22 (43.1%) BSI were caused by *Enterococcus faecalis* and *Enterococcus faecium*, respectively. The cumulative incidence of ICU-acquired enterococcal BSI was of 229 episodes per 1000 ICU admissions (95% mid-p confidence interval [CI] 172–298). Most patients received an empirical therapy with at least one agent showing *in vitro* activity against the blood isolate (38/43, 88%). The crude 30-day mortality was 42% (18/43) and 57% (4/7) in the entire series and in patients with vancomycin-resistant *E. faecium* BSI, respectively. The sequential organ failure assessment (SOFA) score showed an independent association with increased mortality (odds ratio 1.32 per one-point increase, with 95% confidence interval 1.04–1.66, *p* = .021).

**Conclusions:**

The cumulative incidence of enterococcal BSI is high in critically ill patients with COVID-19. Our results suggest a crucial role of the severity of the acute clinical conditions, to which both the underlying viral pneumonia and the enterococcal BSI may contribute, in majorly influencing the outcome.

**KEY MESSAGES**
- The cumulative incidence of enterococcal BSI is high in critically ill patients with COVID-19.
- The crude 30-day mortality of enterococcal BSI in critically ill patients with COVID-19 may be higher than 40%.
- There could be a crucial role of the severity of the acute clinical conditions, to which both the underlying viral pneumonia and the enterococcal BSI may contribute, in majorly influencing the outcome.

## Background

Critically ill patients with acute hypoxemic respiratory failure due to coronavirus disease 2019 (COVID-19) requiring mechanical ventilation in intensive care units (ICU) have been reported to possibly be at increased risk of developing bloodstream infection (BSI) compared with other non-COVID-19 critically ill patient populations [1–3]. Although the exact causal pathways of this increased risk are still not completely clear, different non-mutually exclusive mechanisms have been proposed: (i) the use of immunomodulatory agents; (ii) the impairment of antigen presentation and the presence of acquired immunosuppression due to SARS-CoV-2; (iii) the impairment of microcirculation due to the endothelial dysfunction and coagulopathy occurring during COVID-19 [1,4,5].

Regarding the aetiology of BSI in critically ill patients with COVID-19, an unexpectedly high prevalence of enterococcal BSI has been previously observed [3,6,7]. Against this background and considering the frequent use of antimicrobials in critically ill patients with COVID-19 [8,9], the risk of selecting difficult-to-treat resistant strains such as vancomycin-resistant enterococci (VRE) may be non-negligible [10], in turn possibly impacting patients’ outcomes.

The present, descriptive cases series was aimed to better depict the characteristics of ICU-acquired enterococcal BSI in critically ill patients with COVID-19, especially in the terms of cumulative incidence, causative microorganisms, and outcome.

## Methods

This retrospective, single-centre study was conducted in two ICU wards (up to a maximum of 39 beds for COVID-19 patients, with their number being dynamically reduced/increased according to the local COVID-19 epidemiology) at San Martino Policlinico Hospital, a 1200-bed teaching hospital in Genoa, Italy. From 1 January 2020 to 31 December 2020, all patients with COVID-19 and ICU-acquired enterococcal BSI were included in the study.

The primary objective was to describe the characteristics of ICU-acquired enterococcal BSI in critically ill patients with COVID-19, in terms of cumulative incidence, causative microorganisms, clinical characteristics, antimicrobial treatment, and 30-day mortality. A secondary objective was to exploratorily assess the predictors of 30-day mortality in critically ill COVID-19 patients with ICU-acquired enterococcal BSI.

The collection of anonymized data for the present study was approved by the local Ethics Committee (Liguria Region Ethics Committee, registry number 163/2020) and specific informed consent was waived due to the retrospective nature of the study.

### Definitions

COVID-19 was defined as at least one real-time polymerase chain reaction assay positive for SARS-CoV-2 on a respiratory specimen. ICU-acquired enterococcal BSI was defined as at least one blood culture drawn at >48 h after ICU admission positive for enterococci [11].

### Data collection

The following data were collected from the laboratory database and the patients’ medical records as they were at the time of the first enterococcal BSI episode: age in years; gender; Charlson score [12]; diabetes mellitus; chronic obstructive pulmonary disease; chronic kidney disease [13]; previous myocardial infarction; presence of solid neoplasm; presence of hematological malignancy; solid organ transplant; haematopoietic stem cell transplantation; admission from a long-term care facility; previous hospitalization (within 6 months); previous vancomycin-resistant enterococci (VRE) isolation (within 6 months); acute respiratory distress syndrome (ARDS) at hospital admission (at least mild according to Berlin criteria [14]); need for invasive mechanical ventilation (before the development of enterococcal BSI); previous therapy with glycopeptides (within 6 months); anti-inflammatory treatment for COVID-19 (steroids and/or tocilizumab); ICU stay in days before the development of the first enterococcal BSI episode; recent treatment with cephalosporins (during hospital stay before the development of BSI), presence of neutropenia (defined as an absolute neutrophil count <500 cell/mm^3^); presence of a central venous catheter (CVC); Pitt bacteraemia score [15]; sequential organ failure assessment (SOFA) score [16]; presence of septic shock [17]; causative agent of the enterococcal BSI; presence of a polymicrobial BSI (and type of concomitant aetiological agent other than *Enterococcus* spp., with at least two consecutive cultures positive for the same pathogen being necessary for defining BSI due to coagulase-negative staphylococci or other common skin contaminants); DENOVA score [18]; presence of a CVC-related BSI (CRBSI) [19]; concomitant endocarditis (the presence of IE was defined according to the modified Duke’s criteria [20]); adequate source control, that is, unnecessary, removal of infected devices, or drainage of infected fluid collections; administration of an empirical therapy; administration of an appropriate empirical therapy (defined as administration of at least one agent with *in vitro* activity against the given blood isolate).

### Microbiology

The Vitek MS MALDI-TOF mass spectrometry (bioMérieux, Craponne, France) was routinely used for identifying *Enterococcus* spp. as causative microorganisms of ICU-acquired BSI, whereas the Vitek 2 automated system (bioMérieux, Craponne, France) was routinely used for susceptibility testing. The results of the susceptibility tests were interpreted according to the criteria of the European Committee on Antimicrobial Susceptibility Testing (EUCAST) (breakpoint tables for interpretation of minimum inhibitory concentrations [MIC] and zone diameters, version 10.0, 2020; http://www.eucast.org). For daptomycin, isolates were considered susceptible in the case of MIC ≤2 mg/L [21].

### Statistical analysis

No sample size calculations *a priori* were performed for this descriptive, exploratory study. For the primary descriptive analysis, the cumulative incidence of ICU- acquired enterococcal BSI was calculated as the number of events per 1000 ICU admissions of COVID-19 patients, with exact mid-p 95% confidence interval (CI) [22]. In the case of multiple episodes of enterococcal BSI from the same species occurring in the same patient, a novel event was considered as independent from the previous one if developed at least 30 days after the last positive culture related to the previous episode. With regard to the demographic and clinical characteristics of single patients, categorical variables were summarized with numbers and percentages, and continuous variables with medians and interquartile ranges. The 95% CI was calculated for all estimates [23,24].

For the secondary exploratory analysis of predictors of 30-day mortality, the first ICU-acquired enterococcal BSI per patient was considered. The possible association between clinical variables and 30-day mortality was first tested in univariable logistic regression models. Then, variables potentially associated with the outcome in univariable comparisons (*p* < .20) were included in an initial logistic regression multivariable model, and then further selected for the final multivariable model through a stepwise backward procedure based on the Akaike information criterion. The survival of patients with ICU-acquired BSI was also summarized graphically through the Kaplan–Meier method, with the time of origin set at the day when the first positive culture of the first enterococcal BSI episode was drawn. Statistical analyses were performed using the R Statistical Software (version 3.6.0, R Foundation for Statistical Computing, Vienna, Austria).

## Results

During the study period, 223 patients with COVID-19 were admitted to the participating ICUs. Overall, 51 episodes of enterococcal BSI, occurring in 43 patients, were registered. 29 (56.9%) and 22 (43.1%) BSI were caused by *Enterococcus faecalis* and *Enterococcus faecium*, respectively. The cumulative incidence of ICU-acquired enterococcal BSI was of 229 episodes per 1000 ICU admissions (95% mid-p confidence interval [CI] 172–298). The cumulative incidence of *E. faecalis* and *E. faecium* BSI was 130 (95% mid-p CI 89–184) and 99 (95% mid-p CI 63–147) episodes per 1000 ICU admissions, respectively.

The demographic and clinical characteristics of the study population are shown in Table 1. As shown in the table, the median age of patients with ICU-acquired *Enterococcus* spp. BSI was of 63 years (interquartile range [IQR] 58–69) and 77% were males (33/43). The first episode of ICU-enterococcal BSI mainly occurred late during ICU stay (median 18 days from ICU admission, interquartile range 12–32). Overall, a moderate burden of baseline comorbidities was observed, with a median Charlson score of 3 (IQR 2–5). Most patients received an empirical therapy with at least one agent showing *in vitro* activity against the blood isolate (38/43, 88%).

**Table 1.** Demographic and clinical characteristics of COVID-19 patients with ICU-acquired *Enterococcus* spp. BSI.

Variable	No. of patients^a^	%	95% CI
Demographic variables			
Age in years, median (IQR)	63 (58–69)		59–66
Male gender	33/43	77	62–88
Medical history			
Charlson score, median (IQR)	3 (2–5)		2–4
Diabetes mellitus	9/43	21	11–36
Chronic obstructive pulmonary disease	3/43	7	2–18
Chronic kidney disease	8/43	19	9–33
Previous myocardial infarction	4/43	9	3–22
Presence of solid neoplasm	1/43	2	0–12
Presence of hematological malignancy	1/43	2	0–12
Solid organ transplant	0/43	0	0–7
Haematopoietic stem cell transplantation	0/43	0	0–7
Admission from LTCF	4/43	9	3–22
Previous hospitalisation (within 6 months)	7/43	16	7–30
Previous VRE isolation (within 6 months)	5/43	12	5–25
Previous therapy with glycopeptides (within 6 months)	5/43	12	5–25
COVID-19 pneumonia			
ARDS at hospital admission^b^	40/43	93	82–98
Need for invasive mechanical ventilation^c^	43/43	100	93–100
Anti-inflammatory treatment for COVID-19			
Treatment with steroids^d^	34/43	79	64–89
Treatment with tocilizumab^e^	12/43	28	16–43
Variables at BSI onset^f^			
Duration of ICU stay before BSI onset in days, median (IQR)	18 (12–32)		13–23
Recent treatment with cephalosporins^g^	34/43	79	64–89
Neutropenia (AN*C* < 500 cell/mm^3^)	0/43	0	0–7
Presence of CVC	42/43	98	88–100
Pitt bacteraemia score	6 (4–8)		6–8
SOFA score	8 (6–11)		7–10
Septic shock	21/43	49	33–64
Infection variables^h^			
Aetiological agent			
*Enterococcus faecalis*	24/43	56	41–70
*Enterococcus faecium*	19/43	44	30–59
Ampicillin-resistant *E. faecalis*	0/24	0	0–13
Vancomycin-resistant *E. faecium*	7/19	37	17–61
Polymicrobial BSI^i^	19/43	44	30–59
DENOVA score	1 (0–2)		1–1
CRBSI	31/43	72	57–84
Endocarditis	0/43	0	0–7
Source control			
Performed/unnecessary^j^	40/43	93	82–98
Not performed	3/43	7	2–18
Empirical therapy^k^	41/43	95	84–99
*In vitro* active empirical therapy^l^	38/43	88	75–95

ANC, absolute neutrophil count; ARDS, acute respiratory distress syndrome; BSI, bloodstream infection; COVID-19, coronavirus disease 2019; CRBSI, catheter-related bloodstream infection; CI, confidence intervals; CVC, central venous catheter; DENOVA, long Duration of symptoms/Embolization/Number of positive cultures/Origin of infection unknown/Valve disease/Auscultation of murmur; ICU, intensive care unit; IQR, interquartile range; LTCF, log-term care facility; SOFA, sequential organ failure assessment; VRE, vancomycin-resistant enterococci.

^a^Results are presented as No. of patients/Total of patients unless otherwise indicated.

^b^At least mild according to Berlin criteria [14].

^c^Before the development of enterococcal BSI.

^d^Methylprednisolone 1 mg/kg/die (30/34, 88%), dexamethasone 8 mg/die (4/34, 12%).

^e^Tocilizumab was administered at the dosage of 8 mg/kg (single intravenous infusion or repeated once).

^f^The day when the first positive blood culture for *Enterococcus* spp. was drawn.

^g^During hospital stay before the development of BSI.

^h^Related to the first episode of ICU-acquired enterococcal BSI in each patient.

^i^Coagulase-negative *Staphylococcus* spp. (*n* = 9), *Bacillus cereus* (*n* = 1), *Candida auris* (*n* = 1), *Pseudomonas aeruginosa* (*n* = 1), *Morganella morganii* (*n* = 1), Viridans Group *Streptococcus* spp. (*n* = 1), *Candida albicans* plus *Candida auris* (*n* = 1), *Candida albicans* plus *Staphylococcus aureus* (*n* = 1), coagulase-negative *Staphylococcus* spp. plus *Bacteroides fragilis* (*n* = 1), coagulase-negative *Staphylococcus* spp. plus *Enterobacter aerogenes* (*n* = 1), *Enterobacter aerogenes* plus *Pseudomonas aeruginosa* (*n* = 1).

^j^CVC removal (*n* = 28, performed in all cases within 96 h after collection of the first positive culture), not necessary (*n* = 12).

^k^Vancomycin (*n* = 17), daptomycin (*n* = 10), linezolid (*n* = 3), tigecycline (*n* = 1), daptomycin plus linezolid (*n* = 6), daptomycin plus ampicillin (*n* = 1), daptomycin plus gentamicin (*n* = 1), vancomycin plus linezolid (*n* = 1), vancomycin plus amikacin plus linezolid (*n* = 1).

^l^With at least one agent showing *in vitro* activity against the blood isolate: vancomycin (*n* = 14), daptomycin (*n* = 10), linezolid (*n* = 3), tigecycline (*n* = 1), daptomycin plus linezolid (*n* = 6), daptomycin plus ampicillin (*n* = 1), daptomycin plus gentamicin (*n* = 1), vancomycin plus linezolid (*n* = 1), vancomycin plus amikacin plus linezolid (*n* = 1).

**Figure 1.**
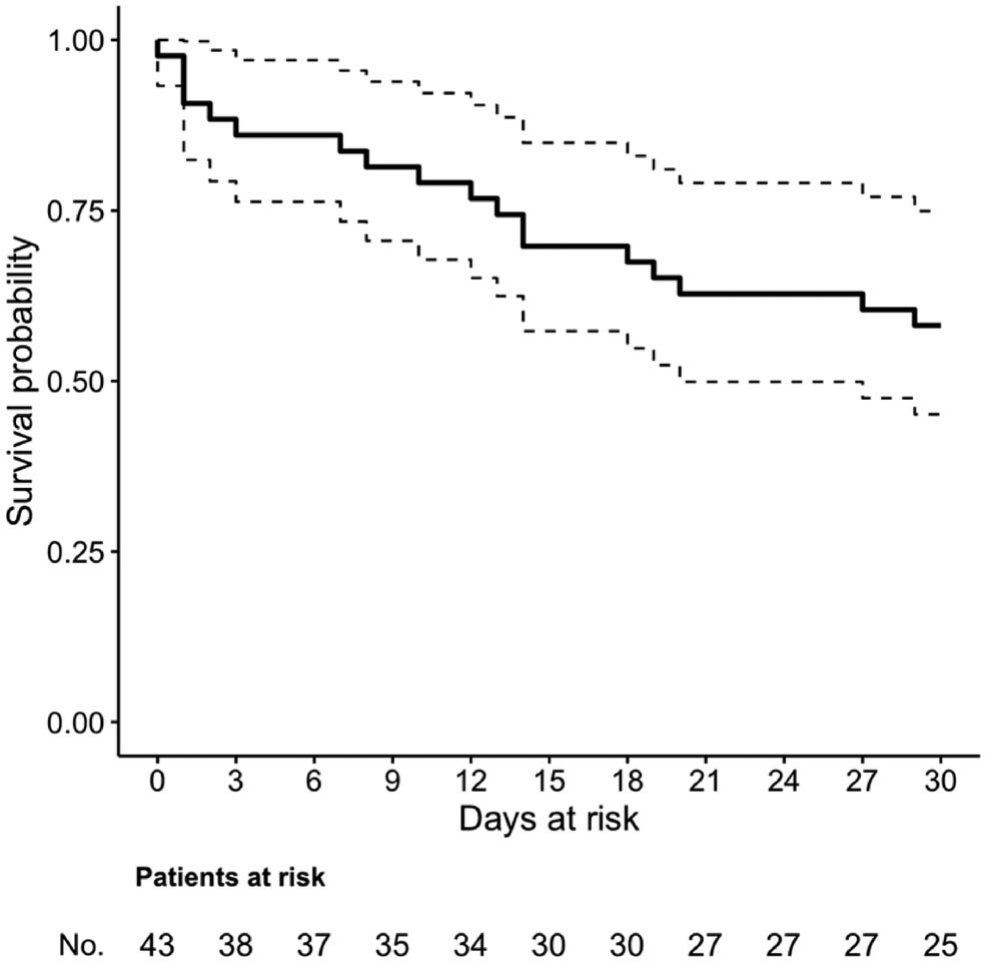
Survival in critically ill COVID-19 patients with ICU-acquired *Enterococcus* spp. BSI. The time of origin was set at the day when the first blood culture positive for *Enterococcus* spp. was drawn.

The crude 30-day mortality in our series was 42% (18/43), as also shown in Figure 1. According to the causative agent of the first enterococcal BSI episode, 30-day mortality was 42% (10/24), 42% (8/19), and 57% (4/7) in patients with *E. faecalis* BSI, *E. faecium* BSI, and vancomycin-resistant *E. faecium* BSI, respectively. The results of the univariable and multivariable analyses of predictors of 30-day mortality are shown in Table 2. In univariable analyses, previous hospitalization and SOFA score showed an association with increased mortality. In the final multivariable model, only the SOFA score retained an independent association with increased mortality (odds ratio 1.32 per one-point increase, with 95% confidence interval 1.04–1.66, *p* = .021).

**Table 2.** Univariable and multivariable analysis 30-day mortality predictors in COVID-19 patients with ICU-acquired *Enterococcus* spp. BSI.

Variable	Non-survivors^a^ 18 (42)	Survivors^a^ 25 (58)	Univariable analysis		Multivariable analysis**	
			OR (95% CI)	*p*-Value	OR (95% CI)	*p*-Value
Age in years, median (IQR)	65 (60–70)	61 (54–67)	1.06 (0.98–1.14)	.150	1.07 (0.98–1.17)	.128
Male gender	14 (78)	19 (76)	1.11 (0.26–4.67)	.892		
Charlson score, median (IQR)	3 (2–5)	3 (1–4)	1.23 (0.87–1.73)	.246		
Diabetes mellitus	4 (22)	5 (20)	1.14 (0.26–5.03)	.860		
Chronic obstructive pulmonary disease	2 (11)	1 (4)	3.00 (0.25–35.91)	.386		
Chronic kidney disease	3 (17)	5 (20)	0.80 (0.16–3.88)	.782		
Previous myocardial infarction	1 (6)	3 (12)	0.43 (0.04–4.52)	.483		
Presence of solid neoplasm	1 (6)	0 (0)	Model not converging	.419*		
Presence of hematological malignancy	1 (6)	0 (0)	Model not converging	.419*		
Solid organ transplant	0 (0)	0 (0)	–	1.000*		
Haematopoietic stem cell transplantation	0 (0)	0 (0)	–	1.000*		
Admission from LTCF	3 (17)	1 (4)	4.80 (0.46–50.50)	.191		
Previous hospitalisation (within 6 months)	6 (33)	1 (4)	12.00 (1.29–111.32)	.029	6.52 (0.62–68.44)	.118
Previous VRE isolation (within 6 months)	3 (17)	2 (8)	2.30 (0.34–15.44)	.391		
Previous therapy with glycopeptides (within 6 months)	1 (6)	4 (16)	0.31 (0.03–3.03)	.313		
ARDS at hospital admission	16 (89)	24 (96)	0.33 (0.03–3.99)	.386		
Need for invasive mechanical ventilation	18 (100)	25 (100)	–	1.000*		
Treatment with steroids	15 (83)	19 (76)	1.58 (0.34–7.38)	.562		
Treatment with tocilizumab	5 (28)	8 (32)	0.82 (0.22–3.09)	.766		
Duration of ICU stay before BSI onset in days, median (IQR)	17 (12–32)	21 (11–32)	1.01 (0.98–1.05)	.476		
Recent treatment with cephalosporins	15 (83)	19 (76)	1.58 (0.34–7.38)	.562		
Neutropenia (AN*C* < 500 cell/mm^3^)	0 (0)	0 (0)	–	1.000*		
Presence of CVC	17 (94)	25 (100)	Model not converging	.419*		
Pitt bacteraemia score, median (IQR)	8 (7–8)	6 (4–8)	1.24 (0.97–1.58)	.086		
SOFA score, median (IQR)	11 (8–13)	7 (6–10)	1.31 (1.07–1.60)	.010	1.32 (1.04–1.66)	.021
Septic shock	10 (56)	11 (44)	1.59 (0.47–5.39)	.456		
VRE as aetiological agent	4 (22)	3 (12)	2.10 (0.41–10.80)	.377		
Polymicrobial BSI	7 (39)	12 (48)	0.69 (0.20–2.36)	.553		
DENOVA score, median (IQR)	0 (1–2)	1 (1–2)	0.92 (0.45–1.86)	.808		
CRBSI	12 (67)	19 (76)	0.63 (0.16–2.42)	.502		
Endocarditis	0 (0)	0 (0)	–	1.000*		
Source control performed/unnecessary	15 (83)	25 (100)	Model not converging	.066*		
Empirical therapy	17 (94)	24 (96)	0.71 (0.04–12.13)	.812		
*In vitro* active empirical therapy	16 (89)	22 (88)	1.09 (0.16–7.31)	.929		

ANC, absolute neutrophil count; ARDS, acute respiratory distress syndrome; BSI, bloodstream infection; COVID-19, coronavirus disease 2019; CRBSI, catheter-related bloodstream infection; CI, confidence intervals; CVC, central venous catheter; DENOVA, long Duration of symptoms/Embolization/Number of positive cultures/Origin of infection unknown/Valve disease/Auscultation of murmur; ICU, intensive care unit; IQR, interquartile range; LTCF, log-term care facility; SOFA, sequential organ failure assessment; VRE, vancomycin-resistant enterococci.

^a^Results are presented as No. of patients/total of patients unless otherwise indicated.

*In the case of zero events in both groups and non-converging univariable logistic regression models, p values are from Fisher exact test. Nonconvergence was also observed when including the variable source control performed/unnecessary (*p* < .20 in univariable analysis) in the multivariable logistic regression model, that was eventually built without this variable. An additional, penalised, multivariable logistic regression model with Firth’s correction, which included source control performed/unnecessary plus all the variables included in the final standard multivariable model, confirmed the results observed in the standard model (age: OR 1.07, 95% CI 0.99–1.18, *p* = .112; previous hospitalisation: OR 2.82, 95% CI 0.40–31.13, *p* = .302; SOFA score: OR 1.26, 95% CI 1.03–1.61, *p* = .025) and no independent association with treatment failure was observed for source control performed/unnecessary (OR 0.23, 95% CI 0.00–3.27, *p* = .296). The additional analysis with Firth’s correction was performed using the logistf package for R Statistical Software version 3.6.0 (R Foundation for Statistical Computing, Vienna, Austria).

**Only results for variables retained in the final multivariable model are presented. The discriminatory performance and the calibration of the model were evaluated using the C-statistic (area under the curve [AUC] 0.835, with 95% CI from 0.702 to 0.967) and the Hosmer–Lemeshow’s test (*p* = .149), respectively.

## Discussion

During 2020, we registered 51 episodes of ICU-acquired enterococcal BSI in 43 different COVID-19 patients, with a high cumulative incidence of 229 episodes per 1000 ICU admissions. A high crude 30-day mortality of 42% was observed, despite the lack of a heavy burden of baseline comorbidities and a high rate of appropriate empirical therapy.

The high cumulative incidence of enterococcal BSI we observed is in line with the results of Bonazzetti and colleagues, who previously registered 55 episodes of enterococcal BSI among 96 critically patients with COVID-19, equal to a cumulative incidence of 573 episodes per 1000 ICU admissions, which was even greater than the high cumulative incidence we observed in our centre [6]. In addition, *Enterococcus* spp., together with coagulase-negative staphylococci, was the most frequently responsible for BSI among critically ill COVID-19 patients in four different previous series [1,3,6,7]. Some possible explanations for an increased predisposition of critically ill COVID-19 patients to develop enterococcal BSI have been proposed. For example, the use of ceftriaxone or ceftaroline (which is inactive against *E. faecium*, although some *in vitro* activity against *E. faecalis* has been reported) as empirical agents in the suspicion of community-acquired bacterial pneumonia superimposed to the viral disease, since this could have exerted a selective pressure increasing the risk of enterococcal BSI [3]. However, large use of cephalosporins may have not been the rule in all series reporting a high cumulative incidence of enterococcal BSI in critically ill COVID-19 patients [1,6]. An alternative, non-mutually exclusive possible explanation is an increased risk of cross-transmission of *Enterococcus* spp. due to possibly relaxed infection-control measures (i.e. during the peaks of the COVID-19 pandemic the protection of healthcare personnel from the virus was prioritized with respect to the allocation of personal protective equipment, with a possible consequent risk of cross-transmission of bacteria across patients [25–27]), although this explanation is not fully in line with the fact that a similar increase in the transmission of several other bacteria (e.g. *Enterobacterales*, staphylococci) was apparently not observed [3,6]. Finally, an intriguing hypothesis is that of an increased bacterial translocation from the gut due to the presence/worsening of intestinal wall damage/inflammation related to the viral infection and/or the host response to the virus [1], which nonetheless deserves further investigation in the light of, again, the apparent absence of a parallel increase in the risk of BSI due to *Enterobacterales* and the lack of a clear elucidation of the underlying causal pathway and mechanisms.

Despite the above-discussed uncertainty in deciphering the true reasons for the high cumulative incidence of enterococcal BSI we and others observed in critically ill COVID-19 patients, the high number of such infections remains, and with the present series, we tried to better depict the characteristics and outcomes of enterococcal BSI in this peculiar population. Notably, despite the high median age of patients in our cohort (63 years), we ultimately registered only a moderate burden of baseline comorbidities (frequency <10% for most of the registered comorbidities, as shown in Table 1), likely reflecting the fact that severe respiratory insufficiency by SARS-CoV-2 may present not only in patients with already existing severe conditions but also in old patients with none or few comorbidities [28]. Another interesting aspect worth discussing is that mortality was high despite most patients (88%) received an *in vitro* active empirical therapy since the onset of BSI, suggesting a possible role of the severity of the underlying viral disease in unfavourably influencing the outcome. Of note, two patients did not receive prompt empirical therapy, possibly due to initially unclear or unrecognized infections in presence of mitigated clinical signs and inflammatory markers at the onset of BSI after previous treatment with anti-inflammatory and immunomodulatory agents [29,30].

The prognostic impact of the patients’ critical conditions is in line with the independent association we observed between higher SOFA score and 30-day mortality in the multivariable logistic regression model, although it should be recognized that this secondary analysis was burdened by the small sample size and the consequent low power for detecting other possible clinically relevant associations. For example, the potential unfavourable impact of vancomycin resistance in *E. faecium*, since, in crude numbers, 30-day mortality was higher in the small subgroup of patients with VRE BSI (57% vs. 42% in the entire series), possibly connected to the fact that 3 of the 5 *in vitro* inactive empirical therapies in the entire series were administered to patients with VRE BSI. Finally, it should be noted that we detected a high number of CRBSI (72%). However, we are unsure whether the CVC was the primary site of infection since we cannot exclude that colonization and infection of the device may have occurred after an initial translocation into the bloodstream from another site (e.g. the gut). Another factor showing a non-statistically significant direction of effect towards increased mortality and deserving further investigation in larger cohorts is the Pitt bacteraemia score (reflecting the severity of BSI presentation), whereas in our cohort there was no apparent association between baseline comorbidities (summarized by means of the Charlson score) and an unfavorable prognosis. The absence of endocarditis diagnoses and the low values of the DENOVA score (reflecting the risk of endocarditis) did not allow us to explore their possible prognostic effect in our cohort.

Besides the low power of the secondary, exploratory analysis of predictors of 30-day mortality (discussed above), the present study has some other important limitations. First, this was a case series of COVID-19 patients with enterococcal BSI, thus no comparison was made with critically ill COVID-19 patients with no BSI or with BSI caused by other microorganisms. However, such a possible comparison was not the aim of our study, which was instead conceived to depict the characteristics and outcome of ICU-acquired enterococcal BSI, once recognized their high cumulative incidence in the target population. Second, owing to the observational nature of the study, which implies the existence of unmeasured confounding, and the small sample size, which precludes more sophisticated adjustment for measurable confounding, the present study was not structured to assess the impact of antibacterial therapy (and the type of employed drug/s) on mortality in a completely reliable way. Other not included data that we were eventually unable to reliably collect retrospectively from all patients were: (i) presence and type of recent chemotherapy; (ii) the time elapsed from the last CVC substitution and development of enterococcal BSI. In addition, we cannot exclude the incompleteness of the information on previous glycopeptide use and previous VRE isolation (e.g. in case of the previous hospitalization in other centers). Finally, the single-centre nature of the study may reduce the generalizability of our findings.

In conclusion, our results are complementary to those of other series reporting a high cumulative incidence of enterococcal BSI in critically ill patients with COVID-19. Our hypothesis-generating findings suggest a crucial role of the severity of the acute clinical conditions, to which both the underlying viral pneumonia and the enterococcal BSI may contribute, in majorly influencing the outcome.

## Data Availability

The data that support the findings of this study are available from the corresponding author upon reasonable request.
